# Beta Thalassemia in Children: Established Approaches, Old Issues, New Non-Curative Therapies, and Perspectives on Healing

**DOI:** 10.3390/jcm13226966

**Published:** 2024-11-19

**Authors:** Raffaella Origa, Layal Issa

**Affiliations:** 1 Department of Medical Sciences and Public Health, University of Cagliari, Ospedale Pediatrico Microcitemico A. Cao, ASL Cagliari, 09121 Cagliari, Italy; 2 Karma Association for Diseased Children and Adolescents, Furn El Chebbak, Beirut VG9G+3GV, Lebanon; layal.issa@karma-leb.org

**Keywords:** beta thalassemia, transfusion therapy, monogenic disorder, serum ferritin, iron overload, anemia, chelation, gene editing, gene therapy

## Abstract

Despite a decrease in prevalence and incidence rates, beta thalassemia continues to represent a significant public health challenge worldwide. In high-resource settings, children with thalassemia have an open prognosis, with a high chance of reaching adulthood and old age with a good quality of life. This is achievable if transfusion therapy is properly managed, effectively mitigating ineffective erythropoiesis and its associated complications while also minimizing excessive iron accumulation. Adequate iron chelation is essential to maintain reactive forms of iron within the normal range throughout life, thus preventing organ damage caused by hemosiderosis, which inevitably results from a regular transfusion regimen. New therapies, both curative, such as gene therapy, and non-curative, such as modulators of erythropoiesis, are becoming available for patients with transfusion-dependent beta thalassemia. Two curative approaches based on gene therapy have been investigated in both adults and children with thalassemia. The first approach uses a lentivirus to correct the genetic defect, delivering a functional gene copy to the patient’s cells. The second approach employs CRISPR/Cas9 gene editing to directly modify the defective gene at the molecular level. No non-curative therapies have received approval for pediatric use. Among adults, the only available drug is luspatercept, which is currently undergoing clinical trials in pediatric populations. However, in many countries around the world, the new therapeutic options remain a mirage, and even transfusion therapy itself is not guaranteed for most patients, while the choice of iron chelation therapy depends on drug availability and affordability.

## 1. Introduction

Hemoglobinopathies, which encompass conditions such as thalassemia and sickle cell disease, are prevalent monogenic disorders worldwide, with an estimated annual incidence exceeding 330,000 affected newborns. These hemoglobin (Hb) disorders contribute to approximately 3.4% of mortality in children below the age of 5 years [1]. Among hemoglobinopathies, beta thalassemia is characterized by the reduced or absent synthesis of beta-globin chains and is currently classified based on transfusion needs into transfusion-dependent (TDT) and non-transfusion-dependent beta thalassemia (NTDT) [2,3].

The epidemiology of this condition has undergone profound changes in recent decades. Migration and population movement have made beta thalassemia a global issue that is no longer limited to malaria-endemic regions of the world. In countries with substantial resources and prevention programs, improvements in management and care have significantly reduced the number of new births per year and greatly increased survival rates, allowing patients to reach adulthood and old age [4].

In developing countries with limited healthcare resources and low awareness of the disease, the prevention of beta thalassemia remains a challenge, and the life expectancy of individuals with thalassemia is not comparable to that of individuals in wealthier nations [5].

While conventional therapy, supported by hundreds of scientific studies, continues to rely on transfusion and iron chelation therapies, and in selected cases, splenectomy [2], new therapies promise to revolutionize the management of beta thalassemia.

New therapeutic approaches are generally classified as curative and non-curative.

In 2022, the first autologous hematopoietic stem cell-based gene therapy indicated for the treatment of adult and pediatric patients with beta thalassemia requiring regular red blood cell (RBC) transfusions was approved in the United States. It is a first-in-class, one-time ex vivo lentiviral vector gene therapy that works by adding functional copies of a modified form of the beta-globin gene into a patient’s own hematopoietic stem cells to enable the production of a modified functional adult Hb (HbA). More recently, the first non-viral, ex vivo CRISPR/Cas9 gene-edited cell therapy for eligible patients with TDT was approved in Europe, the USA, and Saudi Arabia. A patient’s own hematopoietic stem cells are edited at the erythroid-specific enhancer region of the BCL11A gene through a precise double-strand break. This edit results in the production of high levels of fetal Hb (HbF) in RBCs.

None of the non-curative therapies have been approved for pediatric use. In adults, the only drug on the market is luspatercept, which is currently undergoing clinical trials in children.

The costs of innovative therapies, particularly curative but also non-curative, pose substantial challenges to the sustainability and equity of healthcare access, both in wealthier nations and, even more critically, in resource-limited settings.

## 2. Conventional Management Strategies

### 2.1. Initiation and Management of Transfusion Therapy

Transfusion therapy in children with thalassemia is considered appropriate when it enables growth within normal parameters and facilitates a high quality of life by allowing the child to engage in school and play activities comparable to their peers. This therapy should also aim to mitigate ineffective erythropoiesis and its associated complications, as well as minimize excessive iron accumulation.

In accordance with these principles, international guidelines that suggested initiating transfusions for Hb levels < 7 g/dL on two separate occasions have been updated. It is now recommended that regular transfusion therapy be initiated irrespective of Hb levels in the presence of poor growth, vital parameter changes indicative of uncompensated anemia, bone abnormalities, and enlargement of the liver and spleen [6].

According to Origa et al. (2017) [7], this revised approach has resulted in a progressive lowering of the age at first transfusion and higher Hb levels at initiation over the past decade. Notably, only 22.1% of children began transfusions based on laboratory criteria alone, with the majority starting transfusions based on clinical indications.

This is fundamentally due to the growing recognition of the morbidity associated with NTDT and the improved prognosis achieved in transfusion-dependent cases, simplifying the decision-making process for initiating transfusion therapy [8].

Notably, the decision to initiate transfusion therapy cannot be disregarded because of the higher risk of alloimmunization in patients transfused at an older age [9], and with a better understanding of ineffective erythropoiesis, its complications, and interaction with altered iron metabolism [10]. Early and significant expansion of the erythron is currently viewed as an indicator of future exhaustion of compensatory mechanisms for severe anemia [11]. Furthermore, facial deformities are now deemed unacceptable, even for cosmetic reasons.

The IgA assay must be carried out in all children before the start of transfusion therapy. As a general principle in transfusion therapy, IgA deficiency has remained the main indication to wash blood components in order to avoid adverse reactions.

There is also no evidence in support of different pre-transfusion Hb values in pediatric patients compared to those of adults once transfusion therapy is initiated [6,12,13].

It is crucial to emphasize that adherence to international guidelines for initiating and managing transfusion therapy in children is limited to a few countries. In many regions, inadequate or absent transfusion therapy significantly affects the length of life, quality of life, and cardiac function of patients [14,15,16,17].

### 2.2. Management of Iron Status

As in adults, serum ferritin is commonly used to indirectly assess iron status in children with thalassemia. However, its limitations, particularly regarding its elevation, independent of iron overload as an acute-phase protein, have become more obvious in children, who are more prone to recurrent infections.

Generally, each 15 mL/kg transfusion (assuming a hematocrit of 67%) increases the liver iron concentration by one mg/g dry weight [18].

A study involving 125 subjects with hereditary anemias, in which participants underwent 308 magnetic resonance imaging (MRI) studies during their first 10 years of life, revealed an alarming amount of liver iron in very young children, even those below 2 years old, and the presence of pancreatic and cardiac iron loading in children as young as 2.5 years of age. This was observed more frequently in patients with Diamond–Blackfan anemia, congenital dyserythropoietic anemia, and TDT, consistent with the speculation that ineffective or absent erythropoiesis results in high free iron, which then loads through non-transferrin-mediated mechanisms [19].

Based on the results of that study, it is recommended that the management of transfusion-dependent children include MRI evaluation for cardiac, pancreatic, and hepatic iron as soon as possible after diagnosis. However, this recommendation is not consistently followed in clinical practice, and international guidelines report that liver iron concentration and myocardial iron should be monitored regularly in patients from the age of 9 years or younger if they can tolerate MRI scanning without sedation [6].

In a cohort of 77 patients with TDT, aged between 2.5 and 18 years from Cagliari (Sardinia, Italy) and Los Angeles, no patient below 9.5 years of age exhibited detectable cardiac iron. The authors concluded that for cases in which appropriate chelation therapy was administered from birth, cardiac MRI can be postponed until patients are eight years old, as anesthesia is not required at this stage. However, earlier testing is recommended in patients with suboptimal chelation and in those with increased transfusion requirements [20].

In a study of 107 Italian pediatric patients (median age: 14.4 years) with TDT, a high prevalence (21.4%) of significant myocardial iron overload was found using a T2* multiecho technique, which correlated with lower compliance to chelation therapy (*p* < 0.013). Additionally, a pathological liver iron concentration was detected in 77.6% of patients [21].

Other studies have reported different results depending on the country of origin of the patients, availability of chelators, and adherence to chelation therapy. This suggests that a history of chelation strongly influences the likelihood of iron accumulation in pediatric patients [22,23]. Ferritin levels may indicate early organ accumulation, particularly in liver tissue. This finding suggests the need to individualize the age at which MRI is proposed for these patients.

In adulthood, the utilization of pancreatic T2* is not as standardized as it is for cardiac and hepatic T2*. In children with TDT, a recent study reported that pancreatic siderosis is a frequent finding (73%) associated with hepatic siderosis, which represents a risk factor for myocardial siderosis, and alterations in glucose metabolism [24].

Accumulating dangerous levels of iron in the pituitary gland can occur during the first decade of life. Both the pituitary R2 values and pituitary size serve as validated biomarkers of pituitary function. However, the assessment of pituitary gonadotropic function is not feasible until puberty, when irreversible damage may have already occurred, and pituitary MRI is not routinely performed [25].

### 2.3. Principles of Iron Chelation in Children

The debate regarding the optimal time to initiate iron chelation therapy in children with beta thalassemia continues. The first chelating agent used to treat children was desferrioxamine, which became available in wealthier countries in the late 1960s and mid-1970s and was initially administered intramuscularly and later via slow subcutaneous infusion. Despite its inconvenient route of administration, desferrioxamine enabled children with thalassemia to reach adulthood if they adhered sufficiently to the therapy.

**Figure 1:**
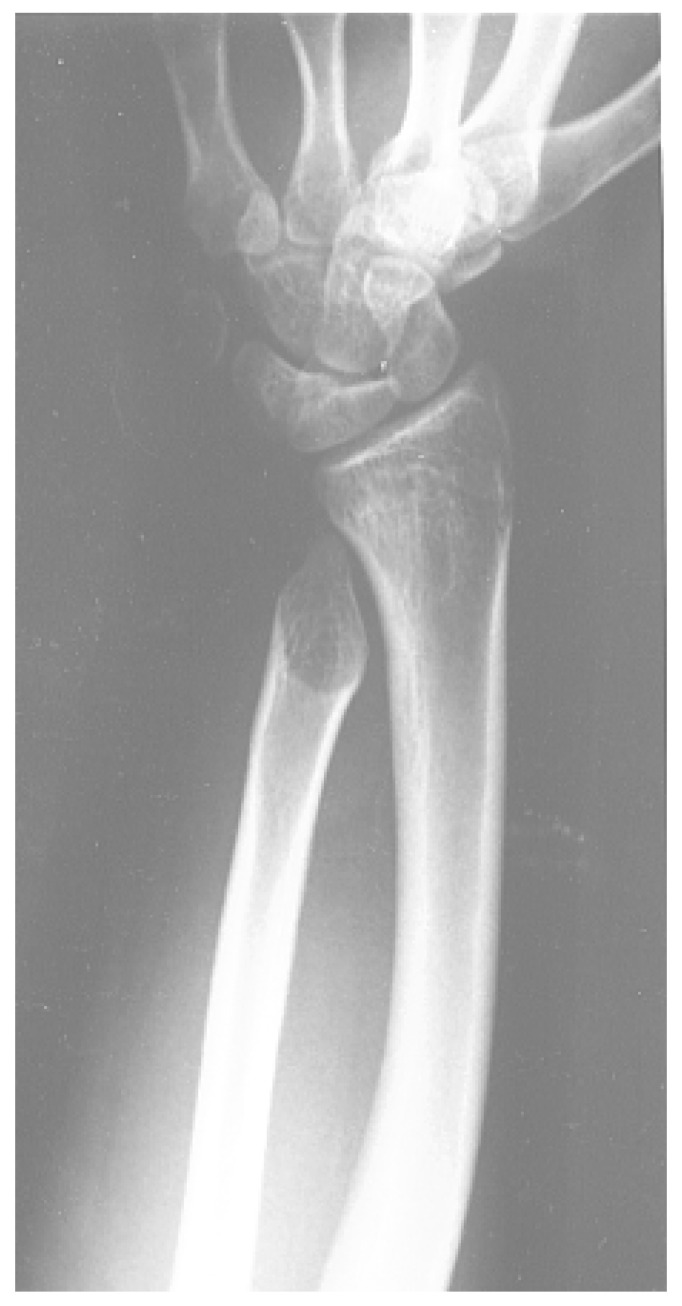
Obvious deformity of the radius and ulna in a woman with beta thalassemia who used desferrioxamine doses of up to 100 mg/kg during the first two years of life.

Between the late 1970s and early 1980s, enthusiasm for a new therapy to prevent death in adolescence and young adulthood combined with the absence of regulations regarding pharmacological experimentation. This led to the use of desferrioxamine at very high doses (up to 100 mg/kg/day) at an early age, in some cases simultaneously with the initiation of transfusion therapy or immediately thereafter. Many cases of toxicity occurred, particularly platyspondyly, metaphyseal alterations (Figure 1), and low stature. Bilateral sensorineural hearing loss for high-frequency sounds is another common complication of using doses of desferrioxamine that are not proportional to the level of iron accumulation [26,27,28,29].

This is also due to the lower chelation selectivity of desferrioxamine compared to chelators that were developed later.

Owing to the risk of desferrioxamine toxicity in early life, international guidelines on the initiation of iron chelation therapy are conservative, recommending initiation with low doses of the chelator [6], after 10–20 transfusions, and when serum ferritin levels exceed 1000 ng/mL. Typically, the average daily dose should range between 20 and 40 mg/kg until skeletal growth is completed.

However, several authors have reported a high probability of iron overload at a young age in transfusion-dependent children, which may lead to early complications and increased toxicity later in life [19,25,30].

Danjou et al. [31] considered labile plasma iron, the primary redox-active and readily chelatable fraction of non-transferrin-bound iron, to be a more direct indicator of impending tissue iron overload. Consequently, they proposed labile plasma iron as a potential marker for initiating chelation therapy. They further suggested that a combination of transferrin saturation and the number of transfused RBCs is the most accurate method for predicting elevated labile plasma iron levels. From a practical standpoint, they recommended initiating chelation therapy when >1000 g of RBCs were transfused or when transferrin saturation exceeded 90% in patients with <1000 g of RBCs transfused [31].

In line with this approach, the present authors regularly monitor both the number of blood units and the volume of transfused blood, as well as transferrin saturation, from the first transfusion onwards. Chelation therapy is initiated after a median of 10 transfusions, once transferrin saturation exceeds 80%.

Moreover, early use of the oral iron chelator deferiprone has been proposed. Compared with desferrioxamine and deferasirox, deferiprone possesses a lower affinity for iron and should therefore be less risky, even in the absence of systemic iron overload, as seen in young children recently diagnosed with thalassemia [32,33].

This is also because deferiprone has an even lower affinity for iron than for transferrin, to which it would release iron, thereby protecting the body from the depletion of iron-bound transferrin that is required for normal iron metabolism [33,34,35].

A randomized, non-placebo-controlled pilot study revealed that the early initiation of deferiprone treatment could safely delay iron accumulation without inducing toxicity [36].

In the subsequent START study, it was further confirmed that early initiation (serum ferritin levels between 200 and 600 μg/L) of oral deferiprone therapy in children with TDT effectively maintains low iron levels. This treatment approach does not appear to be linked to the risk of iron depletion or growth retardation and is generally well tolerated, with a safety profile comparable to that of deferiprone treatment in older patients [37].

Despite the results of the DEEP2 and DEEP3 studies, which demonstrated that deferiprone maintains the same efficacy and toxicity profile across pediatric and adult populations and is non-inferior to deferasirox in pediatric populations (aged 1 month to 18 years) [38,39], this iron chelator is considered a second-line option in Europe. Similarly, prescription information in the United States and many other countries worldwide warns of the scarcity of pediatric data. Consequently, the most commonly utilized pediatric chelator is deferasirox [40], primarily because of its mode of administration, frequency of dosing, and palatability, which has improved with the transition from dispersible tablets to the new film-coated tablet formulation [41,42].

These attributes have clear implications for treatment adherence and preference by young patients [41,43].

The ENTRUST observational study, which evaluated the long-term safety of deferasirox dispersible tablets in pediatric patients (aged ≥2 to <6 years) with transfusional hemosiderosis, exhibited a tolerable safety profile. The study did not reveal any new or unexpected safety concerns, and there were few discontinuations due to adverse events, which decreased further over time [44]. In a single-arm interventional phase 4 study (MIMAS), crushed deferasirox film-coated tablets demonstrated a safety and efficacy profile consistent with previous reports. Overall, patient satisfaction was favorable, and there was an improvement in treatment adherence among children aged <6 years, suggesting that it represents a feasible treatment option for younger patients [45].

More recently, the phase 2 CALYPSO trial recruited 224 patients with transfusional siderosis (TDT: 63.4%; median age: 5 [range 2–16] years) and showed comparable compliance, safety, and efficacy between the deferasirox granule formulation and deferasirox dispersible tablets [46].

Considering efficacy, the initial data on the use of deferasirox in children date back approximately 20 years [47], and no available data support a distinct efficacy profile of deferasirox between children and adults, nor is there evidence supporting variations in the recommended dosing range based on age [48,49,50,51].

As with use in adults and other chelators, determining the initial dose and adjusting the dosage during iron chelation therapy requires consideration of existing organ iron accumulation (if MRI data are available) and/or serum ferritin levels. This helps to determine whether the objective of chelation is to achieve a negative or neutral iron balance. Other fundamental factors to consider when determining the appropriate dose of chelator or its adjustment include previous adverse events and iron input, which depends on transfusion needs [18,52].

Interestingly, children have been found to have a higher iron intake than older individuals. Therefore, all other factors being equal, lower doses per kilogram than those used in adults should not be considered for children. Simultaneously, it is crucial to exercise caution in children with absent or low iron accumulation—who should comprise the majority—since they are particularly at risk for hyperchelation and related adverse events. It is no coincidence that many serious acute events related to deferasirox have been reported in pediatric patients, regardless of whether severe renal, hepatic, or gastrointestinal issues are being considered [51,53,54,55,56,57].

The risk of toxicity also appears to be related to the pharmacogenomics of the drug, which affects pharmacokinetics [58,59,60].

Although deferasirox remains the most commonly used drug in pediatric care, both deferiprone and desferrioxamine continue to hold relevance. This is primarily due to the prohibitive cost of deferasirox in certain regions of the world and the need for individualized therapy even in children. Treatment must be tailored to the specific needs of each patient, taking into consideration factors such as age, side effects associated with prior treatments, and the distribution of iron accumulation as assessed by MRI. Table 1 presents key characteristics of the three drugs in relation to pediatric use, highlighting specific advantages and disadvantages that become apparent in everyday clinical practice.

In selected cases, such as adults, when there is severe and risky iron overload due to previous inadequate compliance with monotherapy and/or intolerance to one or more chelators at functional doses, a combination of two iron chelators can be considered [61].

Pediatric experiences with the simultaneous administration of deferiprone and desferrioxamine, as well as more recent experiences with the combination of the two oral chelators or with the administration of desferrioxamine and deferasirox, do not show distinct differences in efficacy or the possibility of increased side effects compared to those described in adults [62,63,64,65,66,67,68,69].

Albeit heterogeneous, several analyses have also confirmed the efficacy of the combination of deferasirox plus desferrioxamine in reducing serum ferritin and removing hepatic iron in children aged seven and older who had been unresponsive to standard iron chelation therapy [62,70,71,72,73]. Most studies, despite involving only a small number of patients in some cases, have also confirmed a positive effect on cardiac iron. Moreover, the safety of the combination of deferasirox plus desferrioxamine was consistent with monotherapies, and no distinctive warnings have emerged [61,70,71].

### 2.4. Role of Splenectomy in the 2020s

Splenectomy is being performed less frequently in younger generations of patients with beta thalassemia [74]. The absence of the spleen increases the risk of venous thromboembolic events in adulthood. This risk arises from the loss of the spleen’s function in scavenging thrombogenic red blood cells, which expose phosphatidylserine residues on the outer lipid bilayer and behave like activated platelets. Additionally, the increased number of activated platelets in splenectomized patients contributes to this risk [75,76]. The absence of splenic function also poses the risk of overwhelming post-splenectomy infection (OPSI), which is particularly pronounced with encapsulated microorganisms, such as *Streptococcus pneumoniae*, *Neisseria meningitidis*, and *Haemophilus influenzae*. The likelihood of post-splenectomy sepsis is higher in thalassemia than in cases of post-traumatic splenectomy and spherocytosis and is highest when splenectomy is performed before the age of 5 years and during the first year following the procedure. However, the risk persists throughout life. Given the increased risk of OPSI in young individuals, splenectomy is typically avoided before the age of 5 years.

Estimating the current OPSI risk in individuals aged > 5 years is challenging owing to retrospective study limitations and the inclusion of patients with diverse diseases who may not have been fully immunized. The widespread use of conjugated vaccines can reduce the risk significantly. Strategies to mitigate the development of OPSI include patient education, emphasis on prompt action in response to febrile episodes, vaccination, and prophylactic antimicrobial therapy [77]. Comprehensive guidelines for preventing and treating infections in splenectomized or asplenic patients can be found in publications provided by the British Committee for Standards in Haematology [78].

The utility of splenectomy before hematopoietic stem cell transplantation (HSCT) in patients with TDT is debatable. According to Sanpakit et al. [79], it is associated with faster neutrophil engraftment and lower rejection rates but does not achieve significantly better overall survival or affect mortality. The authors concluded that, as splenectomy does not offer distinct advantages, it should not be routinely performed as part of a pre-HSCT regimen for patients with TDT and splenomegaly. Instead, early and adequate blood transfusions are recommended to prevent splenomegaly [79]. Owing to the delayed initiation and inadequate management of transfusion regimens in many countries, which results in the development of splenomegaly and its associated complications, splenectomy continues to be frequently performed in these regions of the world [80]. A recent multicenter study in Thailand identified infections as the most prevalent thalassemia-related complication in 231 pediatric, adolescent, and young adults with TDT. Splenectomy and pre-transfusion Hb were the only risk factors [81]. Similarly, in a cohort of 39 children with TDT from Turkey who underwent splenectomy at an average age of 11.2 ± 3.2 years, long-term complications included thrombosis in 2 patients (5.1%), infections in 11 patients (28.2%), and pulmonary hypertension in 4 patients (10.2%) [82].

According to international guidelines, splenectomy should be considered in patients with TDT (adults and children, with the specification that it is strongly discouraged in patients under the age of 5 years) in the following cases:

– Increased transfusion requirements prevent optimal control of iron overload after ensuring that it is not due to allo/autoantibodies or bleeding. The annual transfusion volume used as a threshold for increased transfusion requirements is 200–275 mL/kg/year; if effective chelation therapy is maintained despite increased transfusion requirements, splenectomy may not be necessary.
– Hypersplenism with cytopenia
– Symptomatic splenomegaly (symptoms such as left hypochondrial pain or early satiety)
– Cytopenias
– Massive splenomegaly with risk of splenic rupture [6]

When the removal of the spleen is necessary, laparoscopic splenectomy is currently considered the gold standard technique for removing a normal-sized or slightly enlarged spleen and is preferred over open splenectomy. Compared with open splenectomy, laparoscopic splenectomy is less traumatic, associated with fewer complications, requires shorter hospital stays, yields better cosmetic outcomes, and is more cost-effective. Several prognostic scores have been proposed to predict children who are more susceptible to complications and improve procedural outcomes [83].

Partial splenectomy can be viewed as a temporary solution. Following a decrease in transfusion requirements, an increase in the size of the remaining splenic tissue may lead to a subsequent increase in transfusion requirements. Therefore, partial splenectomy is typically recommended only in selected cases, particularly in children aged <5 years [84].

## 3. Growth and Puberty

Short stature is frequently observed in individuals with thalassemia, with prevalence rates ranging from 8% to 75%. The causes of growth disorders include chronic anemia, nutritional deficiency, iron overload, and iron chelation therapy. Therefore, it is essential to actively monitor the growth of all children with thalassemia from birth to adulthood, utilize appropriate growth curves relative to the reference population, and consider Tanner staging and genetic target evaluation. Growth assessments should be conducted every six months or at least once per year from patient intake until the completion of height growth and pubertal development to ensure prompt intervention for any growth disorder, as untreated conditions can have irreversible, long-term consequences. The recommended screening method involves thorough clinical and auxological evaluation, including measurements of weight, height, body mass index, height when sitting, growth rate, and Tanner stage [85].

If a growth disorder is identified, re-evaluation of the transfusion regimen, iron chelation therapy, and iron overload status are required. Moreover, investigations of the associated pathologies and micronutrient deficiencies are needed. Collaboration with a pediatric endocrinologist may be necessary for recombinant human growth hormone therapy [85].

However, controversy exists concerning diagnostic cut-offs for growth hormone deficiency, priming with sex steroids, the starting dose for recombinant human growth hormone, and the definition of therapeutic response [86,87,88,89].

Adherence to modern transfusion and iron chelation protocols and avoidance of iron chelator overdose reduced the risk of short stature [90]. The use of oral chelators from a young age, or low doses of desferrioxamine when oral chelators are not feasible, avoids the negative impact of this medication on bone. This limits platyspondyly and other bone complications that primarily occurred in the early 1980s when high doses of desferrioxamine were used disproportionately to the transfusion burden and existing iron overload. Regular chelation prevents hypogonadism and reduces the negative effect of low sex hormone levels on growth hormone production. However, although most well-transfused and chelated children have a height within the genetic target range, the height standard deviation score tends to decrease over time, even in most children receiving oral chelators [90]. A lower prevalence of primary amenorrhea and male hypogonadism has been reported in younger cohorts of patients with TDT, as well as a significant decrease in age at menarche in later cohorts compared to earlier cohorts [74,91,92,93,94].

In 85 women with TDT born between 1965 and 1995, the mean age at menarche correlated with an earlier onset of iron chelation with desferrioxamine, lower serum ferritin levels, body mass index Z score, and a higher prevalence of regular menstrual cycles and duration of menstrual history [93]. Irregular periods (oligomenorrhea and hypomenorrhea) were linked to a later age at menarche (>14 years) and other endocrine complications, suggesting suboptimal adherence to iron chelation therapy [95].

A practical approach to preventing and screening for poor growth in children with TDT, along with a differential diagnosis of its underlying causes, is reported in Table 2.

## 4. Allogenic Hematopoietic Stem Cell Transplantation in Lights and Shadows

In most countries, HSCT remains the only approved curative treatment option for patients with TDT and has been extensively accessible and practiced for decades.

The analysis of 258 transplanted patients (median age at HSCT: 12, range [1,2,3,4,5,6,7,8,9,10,11,12,13,14,15,16,17,18,19,20,21,22,23,24,25,26,27,28,29,30,31,32,33,34,35,36,37,38,39,40,41,42,43,44,45], <16 years of age: 161) matched with a cohort of age- and sex-matched conventionally treated patients showed a 30-year survival rate similar to that expected in conventionally treated patients with thalassemia. Transplant-related mortality (13.8%) was similar to the probability of death from cardiovascular events in patients who underwent conventional treatment (12.2%) [96].

Overall, consensus statements now recommend that patients with a matched sibling donor be offered HSCT at an early age [97]. However, the availability of an HLA-matched sibling donor and transplantation-related risks, such as graft failure and acute and chronic graft-versus-host disease (GVHD), have restricted the widespread adoption of allogeneic transplantation.

The ‘Pesaro criteria’ established in the late 1980s and originally used for patients under 17 years of age conditioned with busulfan 16 mg/kg plus cyclophosphamide 200 mg/kg regimen continue to be valuable in guiding clinicians in the selection of suitable candidates for HSCT and in predicting transplant outcomes. These criteria provide a standardized framework for assessing the risks associated with HSCT based on factors such as the presence of hepatomegaly, portal fibrosis, and history of inadequate chelation therapy [98,99].

Over the past 20 years, the outcomes of HSCT in thalassemia have gradually improved, which is attributable to advancements in disease understanding, enhanced supportive therapies, breakthroughs in immunogenetics, expansion of stem cell reservoirs, and refinement of conditioning protocols.

The largest report regarding the number of patients was from the European Society for Blood and Marrow Transplantation (EBMT) in 2016, which encompasses 1493 patients with thalassemia transplanted between 2000 and 2010, with 70% of them transplanted from HLA-identical donors. Transplantation from an HLA sibling yielded the best results, with an overall survival (OS) of 91 ± 1% and an event-free survival (EFS) of 83 ± 1%. The optimal age threshold for transplant outcomes was approximately 14 years, with an OS ranging from 90% to 96% and an EFS ranging from 83% to 93% when transplants were conducted before this age [100].

Another similarly sized international report, which examined outcomes from 1110 transplanted patients, revealed that OS and EFS were highest for patients aged ≤6 years. Specifically, the 5-year probabilities of OS for patients aged ≤6 years, 7–15 years, and 16–25 years, adjusted for donor type and conditioning regimen, were 90%, 84%, and 63%, respectively (*p* < 0.001). The corresponding probabilities of EFS were 86%, 80%, and 63%, respectively (*p* < 0.001).

In conclusion, it is generally agreed upon that the most favorable outcomes, with survival rates exceeding 90%, can be achieved when HSCT is conducted in patients before complications arising from frequent blood transfusions, such as iron overload, alloimmunization, and adverse effects of chelation therapy, begin to manifest.

While the EBMT report did not detail the patients’ clinical conditions and transplantation events, such as causes of transplant-related mortality, in the study by Li et al. [101], infection (34%), GVHD (24%), and graft failure (13%) were the main causes of death. Other causes of death included veno-occlusive disease (6%), interstitial pneumonitis (3%), organ failure (11%), hemorrhage (7%), secondary malignancy (1%), and other causes (1%). The OS and EFS did not differ between HLA-matched related and HLA-matched unrelated donor transplantations (89% vs. 87% and 86% vs. 82%, respectively) [101].

Diverse strategies beyond utilizing a familial or non-familial, identical, or partially mismatched donor include umbilical cord blood transplantation aimed at reducing both acute and chronic GVHD. However, challenges arise primarily from an insufficient cell dosage, leading to high graft failure rates and delayed hematopoietic recovery [102].

HLA haploidentical family donors represent another potential avenue for expanding the donor pool. Initial reports on T-cell-depleted CD34 donation showed a 27% graft rejection rate and low thalassemia-free survival [103]. Recent advancements have improved graft rejection rates and OS by using T-cell receptor ab1 (TCRab1)/CD191-depleted grafts [103,104]. However, delayed immune reconstitution has been observed, leading to morbidity and mortality. Additionally, post-transplant lymphoproliferative disorders such as autoimmune hemolytic anemia or thrombocytopenia have been reported. Procedure-related mortality and morbidity remained higher than those in matched sibling transplantations. Consequently, donations from HLA haploidentical families are currently limited to clinical trials [96]. A reduced-intensity conditioning regimen based on the use of treosulfan has been proposed, with the understanding that partially mixed chimerism of donor- and recipient-derived stem cells can produce adequate and stable hematopoiesis [105]. Several analyses, and a retrospective analysis on behalf of the EBMT Paediatric Diseases and Inborn Errors Working Parties, including 772 patients from 42 countries, confirmed the effectiveness and safety of both busulfan-fludarabine (BU-FLU) and treosulfan-fludarabine myeloablative conditioning regimens, with no significant differences in the incidence of acute GVHD and extensive chronic GVHD between the groups [106,107,108,109,110]. Lüftinger et al. (2002) speculated that there may be a potential advantage for a treosulfan-fludarabine-based conditioning regimen based on the excellent outcomes related to 2-year OS (94.7%) and 60-day neutrophil engraftment, even with regard to fertility preservation [109].

## 5. New Therapies in Children

### 5.1. New Non-Curative Therapies in Children

Even in countries with higher economic resources, despite considerable advancements in conventional therapy, which have led to more favorable prognoses for children born in recent years, thalassemia still significantly impacts the quality of life, demanding commitment and time. Hence, following an advantageous risk–benefit assessment in adults, novel therapeutic approaches are being investigated for use in the pediatric population [111]. These approaches aim to mitigate ineffective erythropoiesis, thereby reducing transfusion requirements, as exemplified by luspatercept, or modulate iron metabolism, thereby diminishing iron accumulation and toxicity, with secondary effects on erythropoiesis.

Luspatercept is the only erythroid maturation agent approved by the Food and Drug Administration (FDA) and the European Medicines Agency (EMA) that targets the latter stages of RBC development. This fusion protein comprises a modified extracellular domain of the activin type IIB receptor and an Fc portion of human IgG [112]. Although its underlying mechanisms have not been fully elucidated, luspatercept functions as a ligand trap, sequestering endogenous ligands of the TGF-β superfamily and impeding the activation of downstream effectors, including the Smad2/3 signaling pathway. This pathway exhibits excessive activation in diseases characterized by ineffective erythropoiesis, such as myelodysplasia and beta thalassemia. Further evidence from thalassemic mouse models indicates that luspatercept diminishes hemolysis and prolongs erythrocyte lifespan [112].

The efficacy and safety of luspatercept were assessed in the multicenter, randomized, double-blind, placebo-controlled, phase 3 BELIEVE study (ACE-536-B-THAL-001) conducted across 65 sites in 15 countries spanning Australia, Europe, the Middle East, North Africa, North America, and Southeast Asia [113]. The trial enrolled adult patients (median age: 30 years) with anemia attributed to beta thalassemia necessitating red blood cell transfusions (6–20 units/24 weeks) with no transfusion-free period exceeding 35 days. Patients treated with luspatercept achieved a statistically significant reduction in transfusion requirements of ≥33% compared to baseline (primary endpoint), with a decrease of at least 2 units of RBCs for 12 consecutive weeks, at fixed intervals and at any 12-week interval compared to placebo (45.1% vs. 2.7%, *p* < 0.0001). Similar results were observed in terms of a reduction in transfusion requirements of ≥50% compared to baseline and when considering any 24-week interval of treatment compared to baseline. Regarding safety, the most prevalent adverse reactions (each reported in ≥15% of patients) were headache, bone pain, and arthralgia. Hyperuricemia is the most frequently reported adverse reaction ≥ grade 3. The most severe adverse reactions were thromboembolic events, such as deep vein thrombosis, ischemic stroke, portal vein thrombosis, and pulmonary embolism. Treatment discontinuation due to adverse reactions was observed in 2.6% of patients treated with luspatercept [114].

A phase II study is currently being conducted to assess the safety and pharmacokinetics of luspatercept in young patients with TDT, aged ≥6 to <18 years old (Table 1). This study comprises two parts: Part A involved participants aged 12–18 years and consisted of two dose escalation cohorts, each with six participants, followed by a dose expansion cohort of 30 participants. Results from the dose-finding phase of the 2a study showed that the safety profile of luspatercept in TDT pediatric patients is consistent with that observed in adults. No dose-limiting toxicities or grade 3 or 4 treatment-emergent AEs (TEAEs) were reported. No TEAEs led to the discontinuation of luspatercept treatment. Pharmacokinetic analyses and efficacy assessments are currently ongoing. After completing the safety assessment of participants who had completed at least one year of treatment in Part A, Part B will focus on participants aged 6–12 years and include two dose escalation cohorts, each with six participants [115].

In RBCs, pyruvate kinase is a crucial enzyme in the final step of glycolysis, a process that sustains energy levels through ATP production. Mitapivat is a novel, first-in-class, small oral molecule that acts as an allosteric activator of the pyruvate kinase enzyme. It has shown efficacy in significantly enhancing the activity of both wild-type and various mutant forms of erythrocyte pyruvate kinase, leading to increased ATP production and decreased 2,3-diphosphoglycerate levels [116]. This is especially beneficial in thalassemia, where RBCs often experience metabolic stress due to ineffective erythropoiesis and increased turnover. The clinical development of mitapivat for adults with TDT and NTDT is nearing completion, with two successful phase III trials demonstrating its safety and efficacy in terms of blood consumption and Hb response, respectively (Energize T and Energize, clinicaltrial.gov NCT04770779 and NCT04770753) [117].

As observed with pyruvate kinase deficiency, it is likely that following adult studies, one or more clinical trials will be designed to include pediatric populations.

Another drug in development is etavopivat, which acts through the same mechanism as mitapivat but selectively targets the erythroid-specific isoform of pyruvate kinase. It can be administered orally once daily. Etavopivat is currently being evaluated in a phase 2 trial (Gladiolus, clinicaltrial.gov NCT04987489) in patients with TDT and NTDT aged 12–65. The study is currently in the patient recruitment phase.

Other molecules are being studied that could indirectly improve ineffective erythropoiesis by limiting iron availability. Vamifeport is an oral ferroportin inhibitor that acts directly on the ferroportin–hepcidin axis by blocking a ferroportin transporter.

Preliminary data in pediatric patients aged 12 years and older and adults with NTDT (VITHAL, clinicaltrial.gov NCT04364269) confirm the safety and efficacy of the treatment in reducing iron levels; however, they do not indicate significant improvements in hematological markers [118].

Similarly, a phase 2 study has recently been completed, which included patients aged 12 to 18 years and evaluated another promising iron metabolism modulator, sapablursen. This molecule is an antisense oligonucleotide (ASO) targeting the transmembrane protease serine 6 (TMPRSS6). A reduction in the TMPRSS6 level prevents TMPRSS6-mediated suppression of the bone morphogenetic protein (BMP)-SMAD signaling pathway and leads to an increase in hepcidin levels. In this case as well, the compound under investigation did not demonstrate a significant ability to improve the Hb levels or markers of ineffective erythropoiesis, although it confirmed a positive impact on iron metabolism.

For this reason, according to some authors, compounds that modulate this pathway should not be viewed solely as alternatives to erythropoiesis modulators like luspatercept but rather as partners in a combined therapeutic approach [119,120].

### 5.2. New Perspectives on Healing

The limited availability of donors, mortality, and risk of complications from allogeneic HSCT, including GVHD, have caused researchers to focus on the possibility of new curative therapies for patients with thalassemia, particularly gene therapy [121].

In gene therapy, each patient serves as their own donor and receives genetically modified cells, which are corrected after creating space with chemotherapy. The advantages of gene therapy in thalassemia include the absence of the need for an HLA-matched donor and hence no requirement for immunosuppression or risk of rejection or GVHD, as with an autologous transplant. Therefore, it can theoretically be considered for patients at high risk of receiving a transplant from a traditional donor. Similar to autologous HSCT, possible disadvantages of this therapy include the necessity for chemotherapy, potential gonadal toxicity (with possible infertility, which takes on particular significance since hypogonadism due to iron overload is less frequent), and organ toxicity (especially in the liver and lungs, with a considerable risk of increased toxicity in organs already severely damaged by iron overload) [122].

Two approaches are used to treat thalassemia: additive gene therapy and gene editing. With additive gene therapy, hematopoietic stem cells from the peripheral blood are collected by mobilization with granulocyte colony-stimulating factor and plerixafor, followed by apheresis. They are then activated ex vivo with a mixture of recombinant human cytokines, fms-like tyrosine kinase receptor 3, stem cell factor, and thrombopoietin, washed, and transduced ex vivo after myeloablative chemotherapy with busulfan using a lentiviral vector containing the corrected beta-globin gene. Two vectors have been used for additive gene therapy in thalassemia, including betibeglogeneautotemcel, which contains autologous CD34+ hematopoietic stem cells and progenitor cells transduced with the BB305 lentiviral vector encoding beta-globin (βA-T87Q).

As of 30 January 2023, 63 patients (median [range] age: 17 [4,5,6,7,8,9,10,11,12,13,14,15,16,17,18,19,20,21,22,23,24,25,26,27,28,29,30,31,32,33,34,35] years) had received betibeglogeneautotemcel in a phase 1/2 or 3 study and were enrolled in a long-term follow-up study, with a median (range) follow-up of 60.1 (23.8–109.5) months. Phase 3 studies used a commercial drug product manufacturing process. Ninety percent (37/41) of the phase 3 patients achieved and maintained transfusion independence through the last follow-up (up to 6 years). Transfusion independence was defined as a weighted average Hb level of at least 9 g per deciliter from 60 days after the last red blood cell transfusion in patients who had not received red blood cell transfusions for 12 months or more and was not influenced by genotype or age [123]. Among the 23 patients with a non-β0/β0 genotype, transfusion independence occurred in 20 out of 22 patients who could be evaluated (91%), including 6 out of 7 patients younger than 12 years of age [124]. The adverse events encountered were those expected from an autologous transplant and were consistent with those typical of myeloablation with busulfan. Despite positive results, additive gene therapy is only available in the United States and the Arabian Peninsula because of its extremely high cost.

Several CRISPR/Cas9 strategies targeting three globin genes (HBB, HBG, and HBA) are feasible [125]. Current CRISPR-based clinical trials in the treatment of beta thalassemia are focused on γ-globin reactivation and HbF production in hematopoietic stem cells. The rationale that led to authorization by the FDA, EMA, and UK is that in thalassemia, as in sickle cell anemia, morbidity is inversely proportional to HbF levels persisting in the postnatal period. A child with thalassemia at birth, still possessing high levels of HbF, does not require transfusion. Moreover, it has been demonstrated that the persistence of HbF in the postnatal period correlates with a later onset of transfusions.

Silencing of the BCL11 enhancer essential for the HbF → HbA switch at the end of pregnancy using the CRISPR/Cas9 system, the revolutionary “genetic scissors,” results in the resumption of gamma chain synthesis and hence HbF in the patient’s hematopoietic stem cells. The description of the first two patients treated with exagamglogene autotemcel (exa-cel) has demonstrated the feasibility of the system, allowing for the achievement of high levels of Hb, almost entirely HbF. The presence of HbF in 100% of the peripheral RBCs confirms the absence of chimerism with the thalassemic marrow and hence the absence of ineffective erythropoiesis [126]. The data currently published pertain to 54 patients aged between 12 and 35 years, 19 of whom are under 18 years of age and 33 (61.1%) with severe genotypes [β0/β0 or β0/β0-like]. For 45 of them, it was possible to evaluate whether the primary endpoint was achieved (transfusion independence ≥12 consecutive months while maintaining a weighted average Hb ≥9 g/dL assessed starting 60 days after the last RBC transfusion for post-transplant support or transfusion thalassemia management). The primary endpoint was achieved in 42 of the 45 patients (93.3%). The duration of transfusion independence was 22.5 months (range: 13.3–45.1). The three patients who did not achieve the primary endpoint were two adults and one adolescent; two discontinued transfusions after 21.6 and 12.2 months and have been transfusion-free for 8.0 and 12.2 months, respectively, and one stopped transfusion after 14.5 months, as of 18 September 2023. Early and sustained increases in total Hb and HbF were similar in adults and adolescents. The safety profile of exa-cel remained generally consistent with myeloablative busulfan conditioning and autologous transplantation. Quality-of-life (QOL) measures showed clinically significant improvements [127].

**Figure 2:**
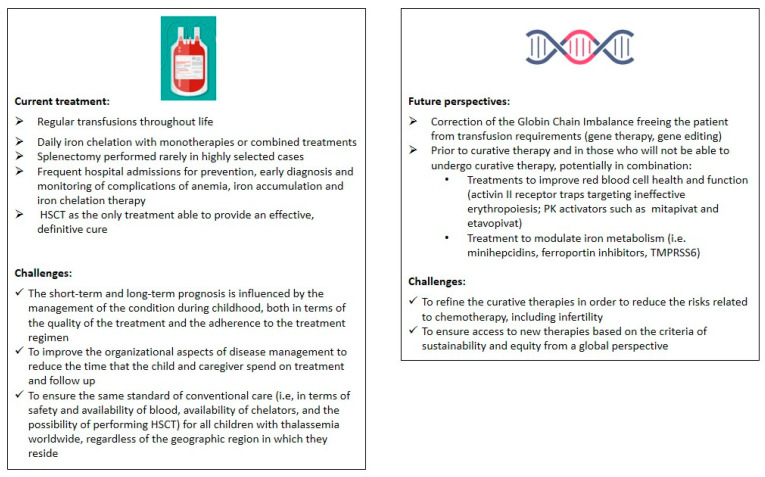
Comparison between conventional therapy and new therapeutic approaches and related challenges. HSCT: hematopoietic stem cell transplantation; TMPRSS6: transmembrane serine protease 6.

Figure 2 compares conventional therapy and new therapeutic approaches, along with the related challenges.

## 6. Management of Thalassemia in Low-Resource Settings

The prevention and management of thalassemia present several challenges in low-resource settings. These include high carrier rates and consanguineous marriages, large variations in management standards and resource capabilities, and the variability of national health insurance coverage within countries (e.g., for nationals vs. expatriates or refugees), which create care gaps that require efforts to facilitate treatment through fundraising programs by treatment centers [5].

Premarital screening is compulsory in some countries but not in others [128]. Diagnosis is mostly made through careful history and physical examination along with examination of red cell indices and is confirmed by Hb electrophoresis or high-performance liquid chromatography, where available. DNA analyses and genotyping are not always available or affordable.

Developing new curative and non-curative therapies poses more sustainability challenges in low-income countries than in high-income countries. Currently, most patients in low-resource settings receive supportive care, including lifelong transfusions and iron chelation therapy.

It is estimated that only 12% of children born with TDT receive adequate transfusion therapy [1]. Blood supply shortages are challenging in low-resource settings, especially in the absence of a national blood bank to supply blood to healthcare facilities. In such cases, it is not always possible to maintain a pre-transfusion Hb level between 9 and 10.5 g/dL, as per international protocols, as the supply of blood depends highly on the recruitment of blood donors by the families, in the absence of a culture of voluntary blood donation. Red cell phenotyping is not always available, which increases the risk of alloimmunization and can limit the availability of compatible RBCs for future transfusions [129]. Another consequence of this policy of obtaining blood for transfusion is that, even in current times, a high percentage of children with thalassemia contract infections transmitted through transfusions, particularly hepatitis C [130].

Serial assessment of serum ferritin every 1–3 months remains the most practical iron overload measurement method because it is inexpensive and readily available, particularly in the absence of MRI techniques to estimate hepatic and cardiac iron levels. This method is used as a standard of care for patient follow-up and treatment decisions. If MRI is available, though not accessible for all patients, Viprakasit et al. recommended prioritizing patients aged 10–18 years with serum ferritin levels >2500 ng/mL, followed by others with ferritin levels above this threshold. This approach aims to optimize chelation therapy and prevent complications. Additionally, patients showing rising serum ferritin levels despite ongoing chelation should be considered for MRI screening. The frequency of follow-up testing should be based on baseline values: annually when cardiac T2* <20 ms and liver iron concentration >7 mg/g dry weight, and every 6 months when cardiac T2* < 10; otherwise, every 2 years [131].

It should be emphasized that when MRI is available, its results guide the frequency of follow-up and therapeutic decisions, making ferritin levels non-determining in this regard.

It is estimated that <40% of transfused children with thalassemia receive adequate iron chelation therapy [1]. The choice of iron chelation therapy in low-resource settings depends on drug availability and affordability. Although generic formulations of deferasirox are becoming increasingly affordable, they are not always accessible in some regions.

Poor adherence to iron chelation therapy is a major challenge in clinical management in low-resource settings, where it can be attributed to issues of accessibility and affordability, in addition to the lack of patient education and awareness of the long-term risks of iron overload.

Although the indications for splenectomy among patients with beta thalassemia are becoming more restrictive, the procedure is still performed to reduce blood transfusion needs in low-resource settings [132]. As expected, splenectomy increases Hb levels and improves the short-term quality of life of children with thalassemia. However, the infectious and thrombotic risks must be considered, leading to the procedure being proposed in ultra-selected cases in high-income countries.

Due to the aforementioned reasons, although general thalassemia management has been improving even in low-income countries, early mortality associated with conservative medical management of thalassemia is high, and suboptimal blood transfusions, iron chelation therapy, and transfusion-transmitted infections continue to be the most significant risk factors. For example, a recent study in India demonstrated that the under-five mortality rate for children with thalassemia is seven times higher (3.5%) compared to the general pediatric population (0.5%). Many of these patients presented with severe anemia and significant hepatosplenomegaly at enrollment, often due to late diagnosis and inadequate management history [133].

Of note, especially in high-prevalence regions of thalassemia, such as South Asia, sex may indirectly affect the epidemiology and health outcomes of thalassemia owing to social, cultural, and economic constraints.

Girls in regions with significant sex disparities often receive less medical care than boys, potentially resulting in more male patients with thalassemia being diagnosed and receiving early treatment at birth, leading to an increased mortality rate among female patients due to delayed diagnosis or lack of systematic treatment [134].

In other words, global inequalities in thalassemia care mirror the economic and social disparities that define the modern world. Rare chronic diseases become even more invisible to national health authorities during times of war or in conflict zones. Identifying actionable solutions to the challenges and barriers to improving access to diagnosis and treatment around the world is not trivial: lobbying and advocacy are required to gain the resources needed or update the pre-existing ones; improving national surveillance mechanisms and registries can serve as a first step towards recognizing these patients’ unmet needs.

Other essential focal points include implementing tools and mechanisms to control treatment costs, identifying and adopting alternative funding mechanisms to address access barriers, ensuring comprehensive care plans are in place to bridge the access gap between rural and urban settings, building capacity and raising awareness among healthcare workers, developing and implementing a patient-centered care model, standardizing clinical guidelines, fostering public awareness, and ensuring patient involvement in decision-making. Achieving these objectives requires the full commitment and engagement of policymakers, as per the Standard Development Goals that share the vision of a world in which health equity is promoted [135].

## Figures and Tables

**Table 1:** Characteristics of the three iron chelators with the indication in beta thalassemia in pediatric use.

	Desferrioxamine (DFO)	Deferiprone (DFP)	Deferasirox (DFX)
Minimum age of use	No minimum age limit	EMA: Limited data available in children under 6 FDA: Three years of age (oral solution); eight years (tablets)	Two years
Indications	First choice for the treatment of iron overload in TDT	FDA: Transfusional iron overload in TDT EMA: In TDT if other chelators are inadequate or not tolerated	FDA: First-line for the treatment of iron overload in TDT EMA: ▪ First-choice for children with TDT from 6 years of age ▪ When DFO cannot be used or is inadequate in children with TDT aged 2 to 5 years who receive frequent blood transfusions (≥7 mL/kg/month of packed red blood cells), in children with TDT from 2 years of age who receive infrequent blood transfusions, and in children with NTDT from 10 years of age
Dosage and frequency	Up to 40 mg/kg 5–7 days per week	▪ 75–100 mg/kg/day in three divided doses daily ▪ 75–99 mg/kg/day twice a day (FDA approved)	▪ Up to 28 mg/kg/day in TDT ▪ Lower doses in NTDT
Main side effects	Local reactions, hearing loss, ocular symptoms, growth retardation and bone toxicity, growth promotion and virulence of *Yersinia* enterocolitica, allergy	Agranulocytosis, neutropenia, gastrointestinal symptoms, joint pain, abnormal liver function tests, decreased levels of zinc	Gastrointestinal symptoms, creatinine increase and reduced creatinine clearance, renal tubulopathy, rash, abnormal liver function tests, gastrointestinal hemorrhage
Advantages with particular reference to pediatric age	▪ The only chelator with extensive clinical experience of the benefits and side effects in children under 2 ▪ An effective alternative for patients with recurrent transaminase elevations that necessitate frequent discontinuation of oral chelators	▪ Oral route ▪ Oral solution suitable for young children and allows for precise dose adjustment ▪ Less likely to cause hyperchelation in comparison with the other chelators	▪ Oral route ▪ Once-daily administration ▪ Limited impact on the daily life of the child and family
Disadvantages and special warnings in pediatric age	▪ Parenteral route ▪ Hyperchelation is possible, especially when MRI is not available, with potentially serious side effects ▪ Growth retardation and bone toxicity are especially common in young children	▪ Multiple daily administrations ▪ The oral solution has poor palatability for many children outside the United States ▪ Frequent blood count monitoring is necessary ▪ Neutropenia and ALT increases; secondary to infections, particularly common in pediatric patients; may be mistaken for side effects of the drug, leading to frequent temporary discontinuation of this iron chelator	▪ Cost ▪ The tablet formulation does not allow for precise dose adjustments, especially in young children ▪ The tablets must be crushed for administration in young children ▪ Hyperchelation is possible, especially when MRI is not available, with potentially serious renal and liver toxicity ▪ ALT increases secondary to infections; particularly common in pediatric patients; may be mistaken for side effects of the drug, leading to frequent temporary discontinuation of this iron chelator

TDT: transfusion-dependent thalassemia; NTDT: non-transfusion-dependent thalassemia; EMA: European Medicines Agency; FDA: Food and Drug Administration; ALT: alanine transaminase; MRI: magnetic resonance imaging.

**Table 2:** A practical approach to preventing and screening for poor growth in children with transfusion-dependent thalassemia and the differential diagnosis of its underlying causes.

Objectives	Strategies	Frequency
Prevention of height and growth disorders	Ensuring a good O_2_ supply: Transfusion regimen started at appropriate * time and continued while maintaining a pretransfusion hemoglobin ≥ 9.5 g/dL	For the entire duration of childhood and adolescence
	Preventing siderosis of liver, heart, pituitary, pancreas, and growth plate; preventing hyperchelation: Iron chelation started at appropriate * time and tailored to the individual patients Compliance to treatment monitored constantly Regular assessments of toxicity signs from iron chelators and serum ferritin Monitoring of iron status with serum ferritin and MRI liver and heart T2*	
	Ensuring that an adequate intake of macro and micronutrients is achieved: Assessment of nutritional status from a clinical perspective and laboratory tests Regular monitoring of vitamin D, zinc, folic acid, and other micronutrients	
Screening of height and growth disorders	Weight, standing and sitting height, BMI, growth velocity Comparing growth data of the patient with the appropriate growth curves and the genetic target	Every 6 months from the start of the patient’s care
	Tanner stage and growth rate to evaluate pubertal development disorders	Every 6 months from the age of 10
Screening of iron-related complications that can negatively impact growth	TSH and FT4 to detect hypothyroidism	Every year from 9 years of age
	Fasting blood glucose or OGTT to detect glucose metabolism disorders	Every two years from 10 years of age
	Serum calcium corrected for albumin value and serum phosphorus	Every year from the age of 10
Investigations for patients with short stature	Reevaluation of transfusion and iron chelation therapy Dietary assessment Laboratory tests: PCR, hepatic and renal function, electrolytes, total proteins, protein electrophoresis, urine analysis, screening for coeliac disease Bone age assessment (X-ray of left wrist and hand) T4, TSH, cortisol, and IGF-1 concentration Refer to endocrinology for assessment of GH secretion	

* Further detailed in Section 2.1 and Section 2.3. BMI: body mass index; OGTT: oral glucose tolerance test; PCR: C-reactive protein test; FT4: free thyroxine; TSH: thyroid-stimulating hormone.

## Data Availability

Not applicable.
